# Supplementary material for: The phased pan-genome of tetraploid European potato
Source: Nature. 2025 Apr 16;642(8067):389–97. doi: 10.1038/s41586-025-08843-0 (PMC12158759; doi:10.1038/s41586-025-08843-0)
Supplement: Supplementary file 5 — Supplementary Figs. 3–14, 20, 21, 23–27 and 34–44. [file 41586_2025_8843_MOESM5_ESM.zip › suppl_figure_34-44/suppl_figure_39_F_v3.pdf]

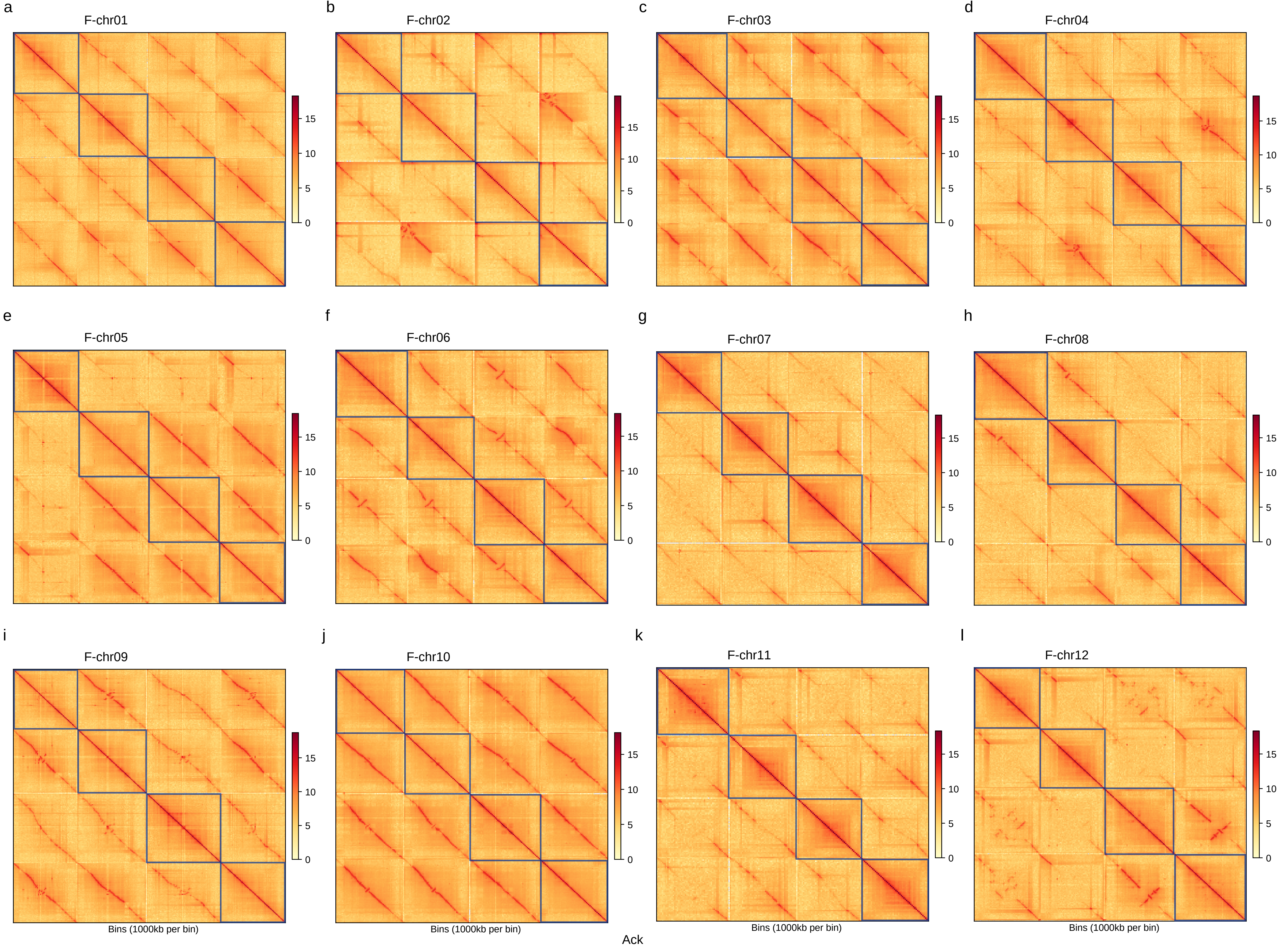
a
F-chr01
b
F-chr02
c
F-chr03
d
F-chr04
e
F-chr05
f
F-chr06
g
F-chr07
h
F-chr08
i
F-chr09
j
F-chr10
k
F-chr11
l
F-chr12
0
5
10
15
Bins (1000kb per bin)

Ack
